# Dynamic Interaction between the CpxA Sensor Kinase and the Periplasmic Accessory Protein CpxP Mediates Signal Recognition in *E. coli*

**DOI:** 10.1371/journal.pone.0107383

**Published:** 2014-09-10

**Authors:** Karolin Tschauner, Patrick Hörnschemeyer, Volker Steffen Müller, Sabine Hunke

**Affiliations:** 1 University of Osnabrück, FB5, Molecular Microbiology, Osnabrück, Germany; 2 Humboldt University Berlin, Department of Biology, Bacterial Physiology, Berlin, Germany; Centre National de la Recherche Scientifique, Aix-Marseille Université, France

## Abstract

Two-component systems, consisting of an inner membrane sensor kinase and a cytosolic response regulator, allow bacteria to respond to changes in the environment. Some two-component systems are additionally orchestrated by an accessory protein that integrates additional signals. It is assumed that spatial and temporal interaction between an accessory protein and a sensor kinase modifies the activity of a two-component system. However, for most accessory proteins located in the bacterial envelope the mechanistic details remain unclear. Here, we analyzed the interaction between the periplasmic accessory protein CpxP and the sensor kinase CpxA in *Escherichia coli* in dependency of three specific stimuli. The Cpx two-component system responds to envelope stress and plays a pivotal role for the quality control of multisubunit envelope structures, including type three secretion systems and pili of different pathogens. In unstressed cells, CpxP shuts off the Cpx response by a yet unknown mechanism. We show for the first time the physical interaction between CpxP and CpxA in unstressed cells using bacterial two-hybrid system and membrane-Strep-tagged protein interaction experiments. In addition, we demonstrate that a high salt concentration and the misfolded pilus subunit PapE displace CpxP from the sensor kinase CpxA *in* *vivo.* Overall, this study provides clear evidence that CpxP modulates the activity of the Cpx system by dynamic interaction with CpxA in response to specific stresses.

## Introduction

Two-component systems (TCS) are the major group of signal transduction systems that allow bacteria to cope with environmental changes. The classical two-component system is composed of a sensor kinase (SK) and a response regulator (RR) [1], [2]. Upon stimulation, the SK becomes autophosphorylated and transfers the phosphoryl group to its cognate RR, which modulates the response [3]. Without stimulation, the response is terminated by dephosphorylation of the RR by either intrinsic activity or by the SK [4]. In the past decade, a number of TCSs were discovered which consist of an additional group of proteins termed accessory proteins [5], [6]. These accessory proteins modulate the response as a co-sensor, scaffolding protein or connector protein and are located in the cytoplasm, the inner membrane or the periplasmic space [6]. How the spatial and temporal interaction of a TCS and its accessory protein modifies the response of a TCS remains unclear for most accessory proteins [6].

The Cpx-envelope stress system of *Escherichia coli* serves as a model to investigate signal integration and signal transduction in TCSs [7]–[9]. It consists of the inner membrane SK CpxA, the cytosolic RR CpxR and the periplasmic accessory protein CpxP [7], [10]. The Cpx-TCS modulates the expression of more than 100 genes important for the integrity of the bacterial envelope, virulence and impacts antibiotic resistance [11]–[16].

A large variety of signals stimulate the Cpx-response. These signals include salt, elevated pH, surface attachment, hormones and stresses that induce protein misfolding in the envelope, resulting in so-called envelope stress [7], [12]. Misfolded envelope proteins accumulate as unordered aggregates and induce bacterial cell death [17]–[23]. CpxP is a Cpx-TCS dependent factor that counteracts extracytoplasmic protein-mediated toxicities [10], [19], hence supporting envelope stress response. Moreover, for misfolded proteins derived from the P pilus of uropathogenic *E. coli* CpxP appears to act as an adaptor protein for the periplasmic protease DegP [19]. On the other hand, *cpxP* overexpression results in a reduced Cpx-response [24], hence interfering with the induction of envelope stress response. Thereby, CpxP inhibits autophosphorylation of reconstituted CpxA [25]. According to the current model the inhibitory and supporting functions of CpxP for envelope stress response are linked: In unstressed cells, CpxP associates with CpxA to shut off the Cpx-TCS. Envelope-stress conditions induce the displacement of CpxP from CpxA resulting in Cpx-TCS activation [19].

This model predicts a direct interaction between CpxP and CpxA. Indeed, several studies provide evidence for an interaction of CpxP with CpxA. First evidence came from the Silhavy group, which showed that tethering an MBP-CpxP fusion protein to membranes of spheroplasts prevents a full Cpx response [26]. Further evidence is provided by structure based functional studies on CpxP [27]. CpxP acts as an antiparallel dimer composed of intertwined α-helices forming a positively charged concave surface [27], [28]. Because the substitution of positively charged residues within the concave surface of CpxP results in decreased inhibition of the Cpx response, it was suggested that CpxP might inhibit CpxA through direct interaction between its concave polar surface and negatively charged residues on the periplasmic sensor domain of CpxA [27]. In support of this suggestion, CpxP inhibits the Cpx response to lesser extent with increasing salt concentrations [27]. Accomplishing peptide arrays indicate that the C-terminal region of the periplasmic sensor domain of CpxA (E_138_DNYOLYLIRPASSSQSDEINLLFD_162_) might play an important role for interaction with CpxP [27]. However, NMR studies could not detect a direct interaction between the periplasmic domain of *Vibrio parahaemolyticus* CpxA (VpCpxA-peri) and the respective CpxP protein (VpCpxP) [29].

Overall, a clear proof of direct protein-protein interaction between CpxA and CpxP is still missing. Here, we show physical interaction between CpxA and CpxP using a bacterial two-hybrid assay (BACTH) [30]. In an alternative approach, membrane-Strep-tagged protein interaction experiments (mSPINE) were used to demonstrate interaction between CpxP and CpxA *in* *vivo* [31], [32]. Additionally, we analyzed the effect of increasing salt concentrations, alkaline pH and misfolded P pilus subunit PapE on the interaction between CpxA and CpxP by mSPINE. Our results show that the interaction between CpxA and CpxP is dynamic and modulated by a high salt concentration and the misfolded pilus subunit PapE.

## Results

### CpxA physically interacts with the accessory CpxP protein

**Figure 1:**
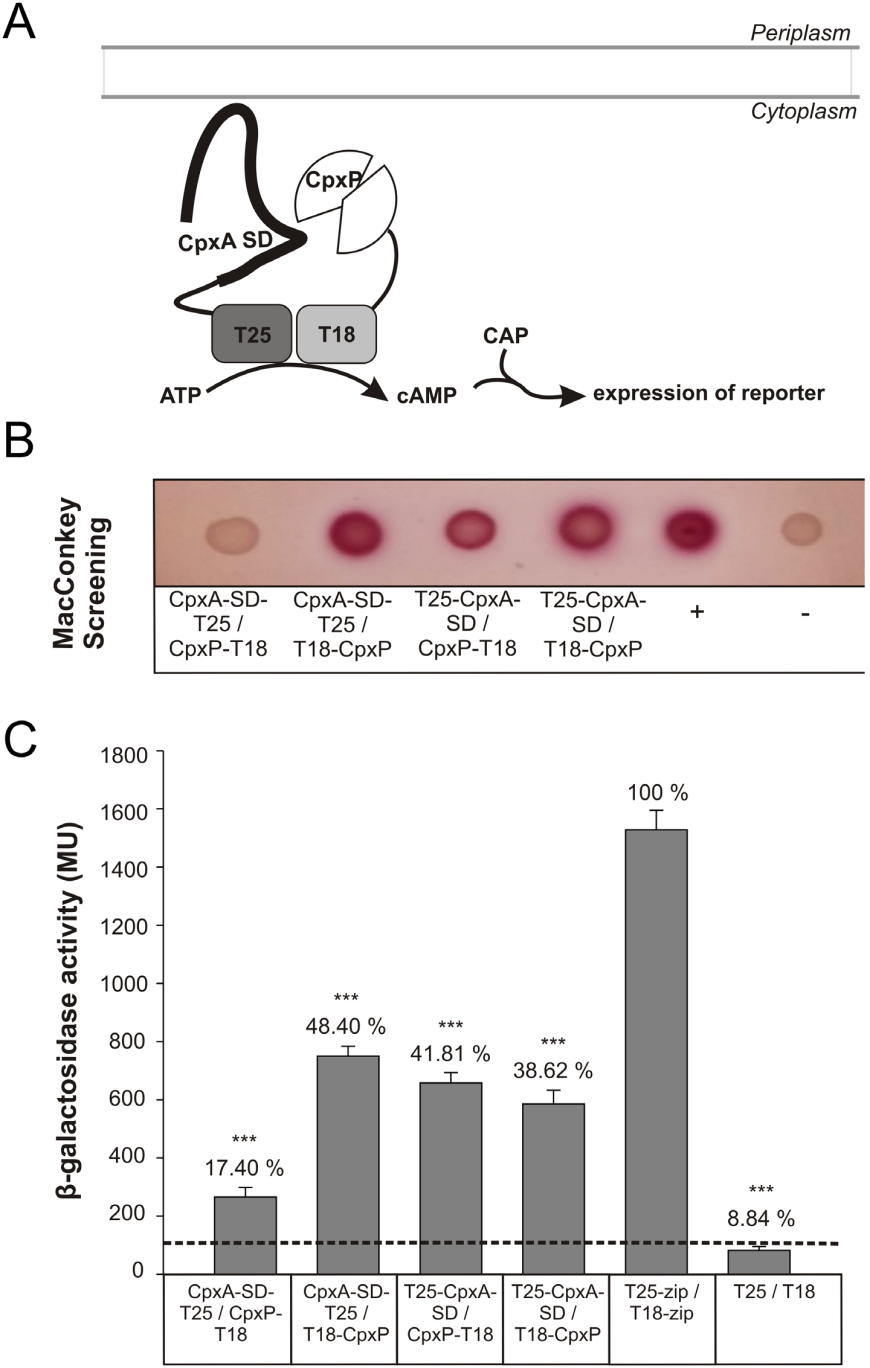
BACTH demonstrates physical interaction between CpxP and the periplasmic sensor domain of CpxA. For protein–protein interaction analysis using a bacterial two-hybrid system (BACTH) CpxP and the sensor domain of CpxA (CpxA-SD) were fused to the N- or C-terminal ends of the T25 and T18 fragments of *B. pertussis* adenylate cyclase as indicated. Strain BTH101 was co-transformed with plasmids encoding the different T25- and T18-hybrid proteins. T25- and T18-fragments fused to the leucine zipper of transcription factor GCN4 and the empty vectors served as positive (+) and negative (−) controls. (A) Illustration of functional complementation of CyaA fragments by BACTH. Interaction between two hybrid proteins in the cytosol results in functional complementation between the T25 and T18 fragments, resulting in cAMP synthesis. cAMP together with the catabolite activator protein (CAP) induces the expression of *E. coli* sugar catabolic operons, such as lactose and maltose. (B) 3 µl of a LB overnight culture were spotted on a MacConkey-Lactose plate and incubated for 24 h at 30°C. (C) The degree of functional complementation between the indicated hybrid proteins was quantified by measuring ß-galactosidase activities in suspensions of toluene-treated *E. coli* BTH101 cells harboring the corresponding plasmids. The activity of the negative control (pKT25, pUT18C) represents the background (dashed line). Shown are the averages ± S.E.M. of three biological replicates each in technical triplicates (t test). Numbers above bars give percentage of ß-galactosidase activity relative to the positive control.

Previous studies suggested that the accessory CpxP protein inhibits the autophosphorylation activity of the SK CpxA by direct interaction [25]. Therefore we initially analyzed whether CpxP interacts directly with CpxA using a bacterial two-hybrid assay (BACTH) [30]. BACTH is based on the functional complementation of the T18 and T25 domains of the adenylate cyclase from *Bordetella pertussis*, resulting in cAMP synthesis (Fig. 1A). For BACTH, we fused the T18 and T25 domains to the sensor domain of CpxA (CpxA-SD) and signal peptide-free CpxP of *E. coli* K12. Consequently, the respective fusion proteins were expected to be expressed in the cytosol. To control that cytosolic expression of the fusion proteins did not confer with correct folding we first confirmed recent crystallographic data showing that CpxP and CpxA-SD form homodimers (Fig. S1) [27]–[29]. Next, the cyclase-deficient E. *coli* strain BTH101 was co-transformed with pairs of fusion plasmids expressing either CpxP or CpxA-SD and the transformants were tested for their ability to metabolize lactose on MacConkey agar plates (Fig. 1B). Co-expression of the fusion proteins CpxA-SD-T25/T18-CpxP, T25-CpxA-SD/CpxP-T18 and T25-CpxA-SD/T18-CpxP showed red colonies demonstrating complementation (Fig. 1B). The efficiency of complementation was quantified by measuring β-galactosidase activities in liquid cultures (Fig. 1C). The β-galactosidase activity generated by the positive control was defined as 100%. Combinations with a red phenotype revealed elevated β-galactosidase activities that were four- to fivefold higher compared to the negative control. A fourfold increased β-galactosidase activity is considered as interaction between the fusion proteins [33]. Thus, our result represents the first direct proof of a physical interaction between the sensor kinase CpxA and its accessory protein CpxP.

### CpxA and CpxP interact *in* *vivo*

To test direct interaction between CpxA and CpxP in the periplasm, membrane-Strep-tagged protein interaction experiments (mSPINE) were performed [31], [32]. mSPINE allows co-purification of reversibly fixed proteins with a Strep-tagged membrane protein. Use of a cross-linker during protein purification enables isolation of transient complexes that are otherwise difficult to detect [34], [35]. Formaldehyde is used as a cross-linking agent which penetrates membranes and allows to snapshot the interactome of living cells [36], [37]. After formaldehyde treatment, cells are harvested and membrane-proteins are purified by detergent treatment and affinity chromatography. Finally, cross-links are cleaved by boiling [37], Strep-tagged membrane proteins are separated from co-purified proteins by SDS-PAGE and protein-interaction-partners are detected by protein-specific immunoblotting [31], [32], [36]. For mSPINE, CpxA of *E. coli* K12 with a C-terminal Strep-tag was cloned into pMal-p2X substituting the maltose binding protein (MalE). This medium-copy vector (∼20 copies per cell) was chosen to ensure that the interaction of CpxA with CpxP was not affected by too high amounts of CpxA-Strep. We tested the localization and function of CpxA-Strep. Therefore, CpxA-Strep was purified as described for mSPINE experiments but without formaldehyde treatment (Fig. S2). As expected, a large amount of protein was found in the so called low speed pellet which contains unbroken cells and aggregated proteins (Fig. S2A). Moreover, CpxA-Strep prepared from the membrane fraction was functional with respect to phosphorylation of CpxR (Fig. S2B).

**Figure 2:**
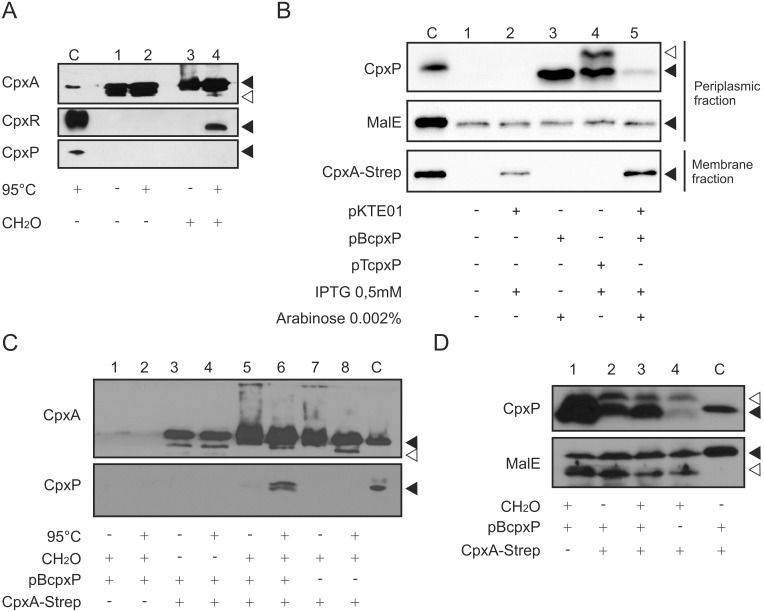
Membrane-SPINE demonstrates physical interaction between CpxP and CpxA in vivo. A) *E. coli* TG1 producing CpxA-Strep (pKT01E) was grown in LB to OD600 = 1.3 and crosslinking was performed for 20 min with 0.6% formaldehyde (CH_2_O). TG1 carrying the CpxA-Strep producing plasmid pKT01E without formaldehyde treatment served as a control. Cytosolic membranes were prepared, membrane proteins were solubilized by detergent treatment and CpxA-Strep was purified according to our established protocol for mSPINE [31], [32]. Aliquots of each sample were incubated at 95°C for 20 min to separate cross-linked proteins from CpxA-Strep and subjected to immunological detection using antiserum to the CpxA, the CpxR and the CpxP protein, respectively. Purified CpxA-His6, His6-CpxR and His6-CpxP served as controls for antibody specificity (C). Black triangles show specific and the white triangle unspecific reactions. Shown are representatives of two biological replicates. B) To check the protein level of CpxP, cell fractionation assays were performed. *E. coli* TG1 cells producing CpxA-Strep (pKT01E) and CpxP from different vectors (pTcpxP, pBcpxP) were grown in LB to OD600 = 0.6. Periplasmic fractions and membrane fractions were prepared and subjected to immunological detection using antiserum to the CpxP protein, the Strep-tag and the MalE protein (loading control), respectively. Purified His6-CpxP, CpxA-Strep and MalE served as controls for antibody specificity (C). C) mSPINE experiments were performed as described in (A) with *E. coli* TG1 producing CpxA-Strep (pKT01E) and CpxP (pBcpxP) grown in LB supplemented with 0.5 mM IPTG and 0.002% arabinose. Cells expressing *cpxA* without a Strep-tag (pEC01E) and *cpxP* (pBcpxP) with formaldehyde treatment (lanes 1 and 2) and cells carrying the CpxA-Strep producing plasmid pKT01E without formaldehyde treatment (lanes 3 and 4) served as controls. Purified CpxA-His6 and His6-CpxP served as controls for antibody specificity (C). Black triangles show specific and the white triangle unspecific reactions. Shown are representatives of two biological replicates. D) To verify similar CpxP protein level in each mSPINE experiment, whole cells from (C) were collected after formaldehyde treatment, and subjected to immunological determination using antiserum to the CpxP protein, and the MalE protein (loading control), respectively. Purified His6-CpxP and MalE served as controls for antibody specificity (C). Black triangles show specific and white triangles unspecific reactions.

Next, we used this CpxA-Strep protein to investigate co-purification of CpxA and CpxR or CpxP. Endogenous CpxR could only be detected after formaldehyde treatment (Fig. 2A, compare lanes 2 and 4). However, an interaction between CpxA and endogenous CpxP was not detected (Fig. 2A, lane 4).

This result was expected, because inhibition of the Cpx-TCS by CpxP can only be monitored after CpxP overproduction [24]. Therefore we used a plasmid with CpxP under control of the arabinose-inducible promoter that allows titration of CpxP production by arabinose (pBcpxP) [27] and is compatible with the CpxA-Strep expressing pKTE01 plasmid. Slight induction of CpxP production from pBcpxP by 0.002% arabinose is sufficient to promote CpxP-dependent degradation of misfolded PapE [27]. In addition, we tested whether overproduction of CpxP from pBad represses the Cpx-TCS. Therefore we assessed the expression of *cpxP* as Cpx-regulated gene using the strain SP594 which carries a chromosomal fusion between the promoter of *cpxP* and the *lacZ* gene. As control, we expressed *cpxP* from a plasmid described to encourage maximum level of Cpx-TCS repression (pTcpxP) (Fig. S3) [24]. β-Galactosidase activity was strongly decreased under all tested CpxP overproduction conditions (Fig. S3A) demonstrating full functionality of CpxP when produced from pBcpxP.

It is known that IPTG inhibits expression from the arabinose promoter [38]. Therefore, we analyzed the extent of inhibition on the production of CpxP from pBcpxP when CpxA-Strep is produced from pKTE01 (Fig. 2B). Without co-expression of CpxA-Strep the amount of CpxP from pBcpxP using 0.002% arabinose is even higher than with pTcpxP (Fig. 2B, compare lanes 3 and 4). However, the amount of CpxP transcribed from pBcpxP drops massively when CpxA-Strep is co-expressed using 0.5 mM IPTG (Fig. 2B, compare lanes 3 and 5). This slight overproduction level of CpxP was sufficient to capture CpxP by CpxA-Strep enrichment (Fig. 2C, lane 6; Fig. S4). Consequently, we used for our further studies strains that slightly overproduced CpxP from pBadcpxP by 0.002% arabinose. Moreover, CpxP was not co-purified in samples without CpxA-Strep (Fig. 2C, lane 2) or without formaldehyde treatment (Fig. 2C, lane 4) emphasizing the importance of formaldehyde cross-linking. Together, our results show direct interaction between CpxA and CpxP for the first time *in* *vivo*.

### Positive charges on the inner cavity of CpxP are important for the interaction with CpxA *in vivo*

**Figure 3:**
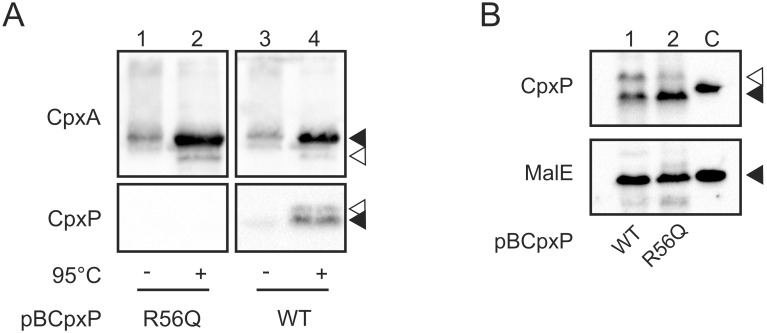
Variant CpxPR56Q is not co-purified with CpxA. A) Substitution of amino acid residue R56 to Q of CpxP results in a stable protein that does not inhibit the Cpx-pathway [27]. mSPINE experiments were performed as described in Fig. 2A with *E. coli* TG1 producing CpxA-Strep (pKT01E) and CpxPR56Q (pBcpxPR56Q/R56Q) or CpxP (pBcpxP/WT) grown in LB supplemented with 0.002% arabinose. Black triangles show specific and the white triangle unspecific reactions. Shown are representatives of two biological replicates. B) To verify similar CpxP protein level in each mSPINE experiment, whole cells from (A) were collected after formaldehyde treatment, and subjected to immunological determination using antiserum to the CpxP protein, and the MalE protein (loading control), respectively. Purified His6-CpxP and MalE served as controls for antibody specificity (C). Black triangles show specific and white triangles unspecific reactions.

From our previous structural study we assume that positive charges located on the inner cavity of the CpxP dimer might be important for the interaction with CpxA [27]. In line with this assumption, substitution of the positively charged amino acids on the inner cavity of CpxP inhibit the Cpx pathway to lesser extent than the wild-type protein [27]. To explore whether substitution of positively charged amino acids on the inner cavity of CpxP impair the interaction with CpxA we performed mSPINE experiments (Fig. 3). mSPINE experiments were performed with cells producing CpxA-Strep and CpxPR56Q (Fig. 3A). In addition, whole cells of each mSPINE experiment were tested by immunoblotting with antibodies targeting CpxP and MalE to verify similar protein expression in all experiments (Fig. 3B). In contrast to wild-type CpxP, CpxPR56Q was almost undetectable (Fig. 3A, lane 2), confirming the assumption that positively charged amino acids on the inner cavity of CpxP are important for the interaction with CpxA [27].

### NaCL disturbs the dynamic interaction between CpxP and CpxA *in* *vivo*

**Figure 4:**
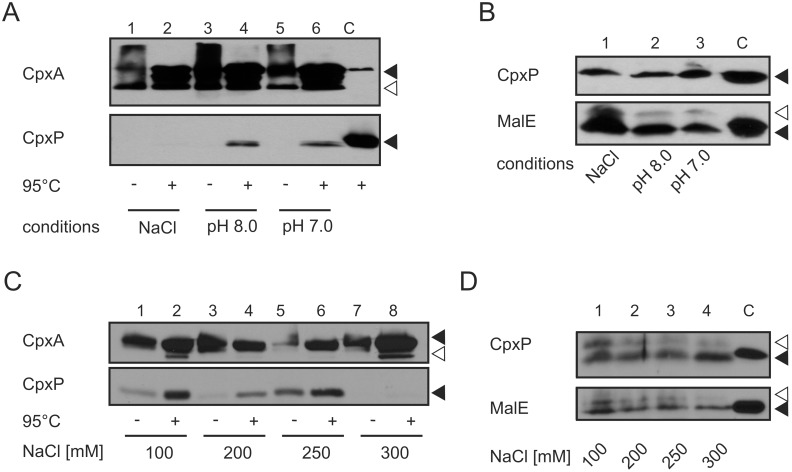
Salt but not alkaline pH disturbs the direct interaction between CpxP and CpxA. A) mSPINE experiments were performed as described in Fig. 2A with E. coli TG1 producing CpxA-Strep (pKT01E) and CpxP (pBcpxP) grown in standard LB (pH 7.0), in buffered medium with alkaline pH (pH 8.0) and in LB adjusted to 0.3 M NaCl (NaCl). Purified CpxA-His6 and His6-CpxP served as controls for antibody specificity (C). Black triangles show specific and the white triangle unspecific reactions. Shown are representatives of three biological replicates. B) To verify similar CpxP protein level in each mSPINE experiment, whole cells from (A) were collected after formaldehyde treatment, and subjected to immunological determination using antiserum to the CpxP protein, and the MalE protein (loading control), respectively. Purified His6-CpxP and MalE served as controls for antibody specificity (C). Black triangles show specific and the white triangle unspecific reactions. C) mSPINE experiments were performed as described in (A) with *E. coli* TG1 producing CpxA-Strep (pKT01E) and CpxP (pcpxP) grown in standard LB (pH 7.0), and in LB supplemented to indicated NaCl concentrations. Purified CpxA-His6 and His6-CpxP served as controls for antibody specificity (C). Black triangles show specific and the white triangle unspecific reactions. Shown are representatives of two biological replicates. D) To verify similar CpxP protein level in each mSPINE experiment, whole cells from (C) were subjected to immunological determination using antiserum to the CpxP protein, and the MalE protein (loading control), respectively. Purified His6-CpxP and MalE served as controls for antibody specificity (C). Black triangles show specific and white triangles unspecific reactions.

Previous functional and structural studies suggest that CpxP might act as a sensor for salt, alkaline pH and misfolded pilus subunits for the Cpx two-component system [19], [27], [28]. According to the current model of Isaac *et al.* [19], CpxP-specific signals alter the interaction between CpxA and CpxP. Upon induction, CpxP is displaced from CpxA resulting in the activation of CpxA [19]. First, we tested whether the environmental stimuli salt or alkaline pH modulate the proposed dynamic interaction between CpxP and CpxA (Fig. 4A). mSPINE experiments were performed with cells grown in LB broth with 300 mM NaCl or in LB broth adjusted to pH 8.0. Standard LB broth served as control (pH 7.0; 150 mM NaCl). In addition, whole cells of each mSPINE experiment were tested by immunoblotting with antibodies targeting CpxP and MalE to verify similar protein expression in all experiments (Fig. 4B, D). CpxP was captured by CpxA-Strep in cells grown in standard LB broth (Fig. 4A, lane 6) and in LB broth adjusted to pH 8.0 (Fig. 4A, lane 4). In contrast, CpxP was almost undetectable in cells grown in LB broth with 300 mM NaCl (Fig. 3A, lane 2), confirming the previous hypothesis that salt disturbs the protein-protein interaction between CpxA and CpxP [27].

Second, in order to determine the NaCl concentration at which CpxP is displaced from CpxA, we performed mSPINE experiments in LB broth with increasing NaCl concentrations. Strikingly, the interaction between CpxA and CpxP was not disturbed up to a NaCl concentration of 250 mM (Fig. 4C, lane 2, 4 and 6). Only for cells grown in LB broth with 300 mM NaCl, interaction between CpxA and CpxP could no longer be detected. Together, our data demonstrate that the interaction between CpxP and CpxA is dynamic and modulated by NaCl.

### Misfolded pilus subunit PapE displaces CpxP from CpxA *in* *vivo*

**Figure 5:**
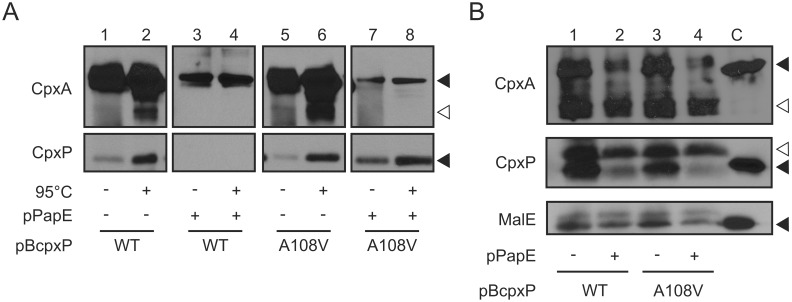
PapE induces the release of CpxP from CpxA. A) mSPINE experiments were performed as described in Fig. 2A with *E. coli* TG1 producing CpxA-Strep and either CpxP (WT) or the cleft mutant CpxPA108V (A108V) without or with PapE co-expression. Due to reduced CpxA-Strep level, mSPINE samples co-expressing PapE were five-fold stronger concentrated than samples without PapE. To allow comparison, immunoblots were cut into an upper and lower part. The upper part was probed with antiserum against CpxA and the lower part with antiserum against CpxP. Immunodetection was carried out for both parts simultaneously. Black triangles show specific and the white triangle unspecific reactions. Shown are representatives of three biological replicates. B) To visualize protein level in each mSPINE experiment, whole cells from (A) were collected after formaldehyde treatment, subjected to immunological determination using antiserum to CpxA, CpxP, and MalE (loading control). Purified CpxA-His6, His6-CpxP and MalE served as controls for antibody specificity (C). Black triangles show specific and white triangles unspecific reactions.

Next, the question was addressed whether the dynamic interaction between CpxA and CpxP is also modulated by misfolded pilus subunits. For this purpose, we used the misfolded P pilus subunit PapE of uropathogenic *E. coli*. P pili assemble via the chaperone-usher pathway which depends essentially on the specific chaperone PapD. Without PapD, PapE misfolds and becomes toxic to cells [18]. CpxP and the periplasmic protease DegP are both needed to counteract the toxicity of misfolded PapE [19]. Based on this observation, it was suggested that misfolded PapE displaces CpxP from CpxA. To test this hypothesis, we performed mSPINE experiments with *E. coli* TG1 cells expressing CpxA-Strep and CpxP in the presence or absence of PapE (Fig. 4A). Cells from each mSPINE experiment served as controls. Note that, the amount of CpxP was drastically reduced when PapE is produced (Fig. 5B). Using mSPINE experiment, CpxP was again verified to interact with CpxA-Strep in cells cultured in standard LB broth (Fig. 5A, lane 2). In contrast, CpxP was not detectable when *papE* was co-expressed (Fig. 5A, lane 4). It is important to note, that the signal of CpxA was significantly lower when PapE was produced, even though these samples were concentrated (Fig. 5A, compare lanes 2 and 4). This lower signal of CpxA in the mSPINE experiment corresponded with a reduced amount of CpxA-Strep in whole cells after overproduction of PapE (Fig. 5B, lane 2). To clarify whether CpxP was not detectable due to the reduced level of CpxA as binding partner, or due to the induced release by PapE, we performed the identical experiments, but using variant CpxPA108V instead of wild-type CpxP. CpxPA108V does not support degradation of misfolded PapE by the DegP protease [27], suggesting that CpxPA108V does not recognize PapE. However, we also observed a drastic reduction of CpxPA108V when PapE is produced (Fig. 5B, lane 4). In contrast, CpxPA108V reduces the Cpx response significantly (Fig. S5A) indicating that the interaction of CpxPA108V with CpxA is hardly affected. Using mSPINE experiment with CpxPA108V, the level of CpxA-Strep was again reduced in whole cells when PapE was produced (Fig. 5A, compare lanes 6 and 8). But in contrast to wild-type CpxP, CpxPA108V was captured by CpxA-Strep when PapE was produced (Fig. 5A, compare lanes 4 and 8). This finding emphasizes that even reduced amounts of CpxA-Strep would be sufficient to capture CpxP. We also proved if a PapE variant that does not activate the Cpx system restores the interaction between CpxA and CpxP. The N-terminal deleted (ntd) PapE variant PapEndt does not activate the Cpx system [39] but restores the interaction between CpxA and CpxP (Fig. S6). Thus, mSPINE allowed us to demonstrate that the misfolded pilus subunit PapE induced the release of CpxP from CpxA.

## Discussion

**Figure 6:**
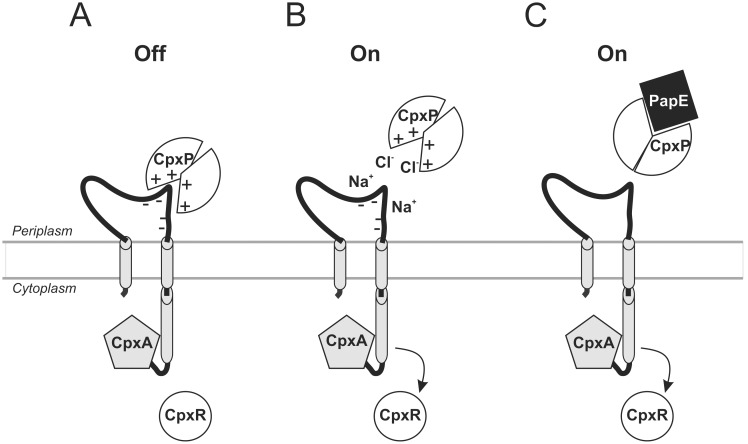
Model depicting CpxP-dependent signal integration by the Cpx-two-component system. (A) Polar interaction between the inner cavity of the CpxP dimer and CpxA keeps the sensor kinase in an “Off” mode. The release of CpxP from CpxA switches CpxA to the “On” mode (B–C). Release of CpxP from CpxA results from a high salt concentration that disturbs the polar interaction between the two proteins (B), or by competing interaction of CpxP with misfolded P-pilus subunits (C) (adapted from [7]).

The mechanistic details of signal integration by two-component systems (TCSs) remain incomprehensible. Especially the function of accessory proteins that act as co-sensors is largely unknown. Our results shed light on the interaction between the periplasmic accessory protein CpxP and the sensor kinase (SK) CpxA in *E. coli*. It was known that CpxP does not only inhibit autophosphorylation of CpxA, but is also essential to counteract toxicity of misfolded pilus subunits in collaboration with the DegP protease [19], [25]. For the first time, we demonstrate physical interaction between CpxP and CpxA in unstressed cells (Fig. 6A). Moreover, using mSPINE we display that this interaction is detached by high NaCl concentration and misfolded pilus subunit PapE. Hence, CpxP modulates CpxA activation by dynamic interaction.

Interestingly, increased NaCl concentration does not result in a gradual release, but in full release of CpxP from CpxA in a single step from 250 to 300 mM NaCl (Fig. 6B). This finding encourages the suggestion that electrostatic interactions might promote the interaction between positively charged residues on the inner surface of CpxP and negatively charged residues on the periplasmic sensor domain of CpxA (Fig. 6A) [27]. The role of electrostatic interactions in protein-protein interactions in general is extensively studied [40]–[44]. Thereby, the amount of electrostatic interactions correlates statistically relevant with the binding strength between two proteins and is modulated by the ionic strength of the medium [40], [43]. Moreover, because CpxA autophosphorylation can be induced by salt independently of CpxP in vitro [25], it is evident that the induced release of CpxP from CpxA by high NaCl concentration is not the only mechanism of the Cpx system to monitor NaCl concentration in the environment. However, the biochemical nature of other mechanisms is so far unclear.

In contrast to increasing NaCl concentrations, alkaline pH does not result in the detachment of CpxP from CpxA. This finding specifies a hypothesis of the Raivio group [28]. Biophysical data showed that CpxP “undergoes a subtle structural rearrangement in response to alkaline pH” suggesting that this slight conformational changes might influence the conformation of a binding partner [28]. Moreover, CpxA autophosphorylation can be induced by alkaline pH independently of CpxP [25]. The combined data implicate that CpxA senses alkaline pH independently from CpxP and that a binding partner different than CpxA is influenced by the conformational change CpxP undergoes in response to alkaline pH.

Similar to a high NaCl concentration, we showed that misfolded pilus subunit PapE detaches CpxP from CpxA (Fig. 6C). Because CpxP and DegP are both essential for PapE degradation, it was concluded that CpxP and DegP work in concert to degrade misfolded PapE [19]. Furthermore, based on the observation that the CpxP protein level is hardly detectable in cells expressing misfolded pilus subunits or grown in alkaline medium in dependency of DegP, it was suggested that CpxP acts as an adaptor that is degraded with its substrate by DegP [19], [45]. In contrast, we monitored only marginal reduction of CpxP protein level when bacteria were grown in alkaline medium and reduced but still visible CpxP protein level after overproduction of misfolded pilus subunit PapE. The discrepancy between the observations might be caused by different approaches to induce *cpxP* overexpression. In order to induce *cpxP* overexpression, we introduced an additional copy of the *cpxP* gene under control of an arabinose-inducible promoter from a plasmid. In contrast, Isaac *et al.* [19] induced *cpxP* overexpression by the use of the strongest cpxA* allele examined so far, *cpxA24*, which constitutively activates the Cpx response and consequently induces also *degP* expression. The *cpxA24* mutation was identified as suppressor of a toxic tripartite fusion protein (LamB-LacZ-PhoA) and a LamB mutant defective in processing (LamBA23D) [21]. The *cpxA24* mutation encompasses a deletion of 32 amino acids in the periplasmic domain (Δ93–124) [46] that corresponds with strands β2 and β3 and the enclosed helix α3 of the PAS domain of the *Vibrio parahaemolyticus* CpxA protein [29]. Interestingly, degradation of the toxic tripartite fusion protein was independent of DegP for the *cpxA24* mutant *E. coli* strain, suggesting that the *cpxA24* mutant features extreme pleiotropy which “regulates additional targets relevant to suppression of the toxic proteins in addition to *degP*” [21].

Hence, our data support the model that the release of CpxP from CpxA is important for CpxP-dependent activation of the Cpx system [19]. Nevertheless, it is evident that modulation of CpxP protein level by different mechanisms is important to fine-tune the Cpx response. Interestingly, expression of *cpxP* is fivefold induced in a *degP* strain [19]. Moreover, the amount of CpxP is reduced not only in stressed but also in unstressed cells in dependency on DegP suggesting post-transcriptional mechanisms to be important to regulate the quantity of CpxP [19]. In line with this suggestion, the signal sequence of CpxP impairs the CpxP level in the periplasm most likely by inefficient translational of the *cpxP* transcript [47].

## Conclusion

Overall, this study emphasized a model in which not only the quantity of CpxP, but also the dynamic of CpxP in the interaction with CpxA modulates the response of the Cpx TCS to periplasmatic stresses. In unstressed cells, CpxP interacts directly with CpxA and blocks CpxA autophosphorylation. As shown here, this interaction between CpxA and CpxP is disturbed by the specific stresses NaCl and misfolded pilus subunits, resulting in CpxA autophosphorylation [25] and subsequently in Cpx response. For a number of pathogens, including *Legionella pneumophila*, *Salmonella enterica*, uropathogenic *E. coli* (UPEC), Shigella spp. and *Yersinia pseudotuberculosis*, the Cpx-TCS modulates the virulence potential [48]–[52]. However, all but one study focussed on the function of the SK CpxA and the RR CpxR for virulence and not on the accessory protein CpxP. Results obtained for *cpxP* UPEC strain indicate that CpxP might be important to colonize specific niches [48]. Further studies are needed to understand on the one hand the function of CpxP for virulence and on the other hand to characterize the interaction sites between CpxP and CpxA and misfolded proteins. These studies will not only help to unravel the function of CpxP but also of accessory proteins in TCS signalling in general.

## Materials and Methods

### Bacterial strains

*E. coli* strain MG1655 [53] served as parent strain for the PCR based amplification of the *cpxA* and *cpxP* genes. *E. coli* uropathogenic strain CFT073 [54] was used to amplify the *papE* gene. *E. coli* strain DH5α (NEB) served as a carrier for the described plasmids. *E. coli* strain BTH101 (Euromedex) was used for bacterial two-hybrid (BACTH) protein interaction studies. *E. coli* strain TG1 [55] was used for *in* *vivo* protein-protein interaction studies and *E. coli* strain SP594 [18] for β-Galactosidase assays. A list of all strains is given in Table 1.

**Table 1:** *E. coli* strains and plasmids used in this study.

Strain/Plasmid	Relevant Gentotype	Reference or Source
MG1655	F- lambda- *ilvG*- *rfb*-50 *rph*-1	[53]
CFT073	Urosepsis isolate (O6:K2:H1)	[54]
DH5α	*fhuA2 lac(del)U169 phoA glnV44 Φ80' acZ(del)M15l gyrA96 recA1 relA1 endA1 thi-1 hsdR17*	NEB
BTH101	F-, *cya-99*, *araD139, galE15, galK16, rpsL1 (Str r)*, *hsdR2, mcrA1, mcrB1*	Euromedex
TG1	F’(*traD*36 *proAB* + *lacI* ^q^ lacZΔM15) *supE thi-1 Δ(lac-proAB) Δ(mcrB-hsdSM)5, (r_K_^−^m_K_^−^)*	[57]
SP594	MC4100, lRS88 [*cpxP*–*lacZ*])	[18]
pKT25	vector generating N-terminal T25 fusion, Kan^R^	Euromedex
pKNT25	vector generating C-terminal T25 fusion, Kan^R^	Euromedex
pUT18	vector generating N-terminal T18 fusion, Amp^R^	Euromedex
pUT18C	vector generating C-terminal T18 fusion, Amp^R^	Euromedex
pKT25-zip	T25-Zip expression plasmid, pKT25 derivative	Euromedex
pUT18C-zip	T18-Zip expression plasmid, pUT18C derivative	Euromedex
pCpxA-SD-T25	CpxA-SD on pKNT25	this study
pT25-CpxA-SD	CpxA-SD on pKT25	this study
pCpxP-T18	CpxP without signal peptide on pUT18C	this study
pT18-CpxP	CpxP without signal peptide on pUT18	this study
pBad18	vector, Amp^R^	[58]
pBad33	vector, Cam^R^	[58]
pIVEX2.1	vector generating C-terminal Strep-tag fusion, Amp^R^, T7-Promoter	Roche
pMal-p2X	vector, Ptrc-Promoter, Amp^R^	NEB
pTrc99A	vector, Ptrc-Promoter, Amp^R^	GE Healthcare
pBcpxP	= pSHE102; CpxP on pBAD33	[27]
pBcpxPA108V	= pSHE102A108V; CpxPA108V on pBAD33 (hydrophobic cleft)	[27]
pBcpxPR56Q	= pSHE102R56Q; CpxPR56Q on pBAD33 (polar concave surface)	[27]
pEC01E	CpxA on pBad18	this study
pI1cpxA	CpxA on pIVEX2.1	this study
pKT01E	CpxA-Strep on pMal-p2X without MalE	this study
pKT02E	PapE on pKT01E	this study
pKT02Endt	PapEndt on pKT01E	this study
pTcpxP	= pSHE100; CpxP on pTrc99A	[27]

### Construction of plasmids

For protein interaction studies using BACTH [30], the target proteins CpxA and CpxP were separately fused to the N or C termini of the T25 and T18 domains. The sensor domain of CpxA (P28–P164) and CpxP without its signal peptide (A21-Q166) were amplified from *E. coli* MG1655 and cloned into the KpnI and XbaI sites of the pKT25, pKNT25, pUT18 and pUT18C vectors. The plasmids pCpxA-SD-T25 and pT25-CpxA-SD were constructed by amplifying the sensor domain of CpxA with the primer pair ECpxA-P28-for and ECpxA-P164-rev and cloned into pKT25 and pKNT25. The plasmids pCpxP-T18 and pT18-CpxP were obtained by amplification of *cpxP* with the primer pair ECpxP-A21-for and ECpxP-Q166-rev and cloning of the amplification products into pUT18 and pUT18C. The T18-*zip* and T25-*zip* control gene fusions were furnished from the BACTH system kit (Euromedex). A list of all constructs is given in Table 1. All oligonucleotides used in this study are listed in Table 2.

**Table 2:** Oligonucleotides used in this study.

Oligo	Sequence
ECpxA-P28-for	5′-TATATCTAGAGCCCAAGCTCGATTCACG-3′
ECpxA-P164-rev	5′-TATAGGTACCAACGGGCGGTCAAACAG-3′
ECpxP-A21-for	5′-TATATCTAGAGGCTGCTGAAGTCGGTTCAGG-3′
ECpxP-Q166-rev	5′-TATAGGTACCAACTGGGAACGTGAGTTG-3′
CpxA5	5′-ATCATATGATAGGCAGCTTAACCGCG-3′
CpxA3	5′-ATCCCGGGACTCCGCTTATACAGCGGCAACC-3′
UPapE-fw	5′-ATATGTCGACGTTTGACAGCTTATCATCGAC-3′
UPapE-rev	5′-AGAACCCCCCGAATATGATGCAACCAG-3′
UPapEntd-fw	5′-ATATAAGCTTATGTGTCTTCCGGTAATGCTGGG-3′
UPapEntd-bw	5′-ATATGTCGACTTACGAATATGATGCAACCA-3′
CpxAFPet-fw	5′-TATAGAGCTCATGATAGGCAGCTTAACCGCGCGCATCTTC-3′
CpxAXbaI-rev	5′-TATATCTAGATTAACTCCGCTTATACAGCGGCAACCAAATCACCAGCCG-3′

For *in* *vivo* protein-protein interaction studies using mSPINE [31], [32], full-length *cpxA* with a C-terminal Strep-tag was cloned into pMal-p2X (NEB). Therefore, the *cpxA* coding region was amplified from MG1655 using primers CpxA5 and CpxA3 and cloned into the NdeI and SmaI sites of pIVEX2.1 (Roche), resulting in pI1cpxA. The obtained plasmid pI1cpxA was digested with NdeI and BamHI and *cpxA-Strep* cloned into the appropriate sites of pMal-p2X, resulting in pKT01E. For overproduction of CpxA without Strep-tag, full-length CpxA was amplified with the primer pair CpxAFPet-fw and CpxAXbaI-rev from *E. coli* MG1655 and cloned into the SacI and XbaI sites of pBad18, resulting in pEC01E. In addition, we constructed a plasmid overexpressing *papE*. Therefore *papE* together with the promoter region of pTrc99A was amplified from pSHE101 [27] with the primer pair UPapE-fw and UPapE-rev and cloned into the HindIII and SalI sites of pKT01E, resulting in pKT02E. For overproduction of CpxP and CpxPA108V, the plasmids pBcpxP and pBcpxPA108V were used [27]. PapEntd was amplified with the primer pair UPapEntd-fw and UPapEntd-bw from pSHE101 and cloned into the HindIII and SalI sites of pKT01E resulting in pKT02Endt.

### Protein interaction studies using BACTH

To estimate the interaction between CpxA and CpxP, BACTH (bacterial two hybrid assay) was performed as described in Karimova *et al*. (1998; 2005). BTH101 *cya* cells were transformed with the recombinant plasmids pKT25, pKNT25, pUT18 and pUT18C in different combinations, which carry either fusions with the gene sequence for the sensor domain of CpxA or the signal peptide free CpxP. The transformants were selected on LB agar plates supplemented with 100 µg ml^−1^ ampicillin, 50 µg ml^−1^ kanamycin and 0.5 mM isopropyl-β-D-thiogalactopyranoside (IPTG) and incubated for 24 h at 30°C. For detection of lactose metabolizing clones, bacteria were grown overnight at 30°C in LB medium containing the appropriate antibiotics. Subsequently 3 µl of the bacterial culture were transferred directly onto MacConkey agar and incubated for 24 h at 30°C. As a positive control, BTH101 cells were transformed with pKT25-zip and pUT18C-zip plasmids and as a negative control, BTH101 cells were transformed with pKT25 and pUT18C plasmids.

For quantitative analysis, β-Galactosidase assays were performed in *E. coli* BTH101 as described [56] with three biological replicates each with technical triplicates.

### Protein interaction studies using mSPINE

*In vivo* protein-protein interaction studies using mSPINE (membrane-Strep-tagged protein interaction experiment) were performed as described [31], [32] with some minor modifications. In brief, *E. coli* TG1 was transformed with plasmid pKT01E or co-transformed with plasmids pKT01E and pBcpxP, pEC01E and pSHE102, pKT02E and pBcpxP, pKT01E and pBcpxPA108V, pKT02E and pBcpxPA108V, as well as pKT02Entd and pBcpxP. Cells were grown in 500 ml LB broth or high salt media (LB broth with 200, 250 or 300 mM NaCl) supplemented with appropriate antibiotics at 37°C. *CpxP* expression was induced with 0.002% arabinose from beginning of cell growth. Overproduction of CpxA-Strep was induced with 0.5 mM IPTG at an optical density of OD_600_ 0.3. After two further duplications, cells were treated with 0.6% formaldehyde and incubated at 37°C for additional 20 min. Subsequently, cells were harvested (8000×g for 10 min) and suspended in 10 ml buffer P1 (20 mM Tris-HCl, 0.5 M sucrose, pH 8.0). Spheroplasts were generated by addition of 1 ml P2 (2 mg/ml lysozyme in 0.1 M EDTA, pH 7.5). Spheroplasts were collected by centrifugation (10,000×g for 30 min) and stored at −20°C overnight. The next day, spheroplasts were resuspended in 6 ml buffer P3 (20 mM Tris-HCl, 0.1 mM PMSF, pH 8.0) and disrupted by sonication (four pulses, each for 1 min) on ice. Unbroken cells and cell debris were removed by centrifugation (10,000×g for 15 min). The supernatant was ultracentrifuged (100,000×g for 30 min) to pellet the membrane fraction. 1 ml of membrane proteins (5 mg/ml) were solubilized in buffer P3 supplemented with 2% dodecyl maltoside (DDM, Glycon, Luckenwalde, Germany) and stirred for 1 h on ice. Subsequently, non-solubilized proteins were separated from solubilized membrane proteins by ultracentrifugation (100,000×g for 30 min). Solubilized membrane proteins were loaded onto a Strep-tactin column (1 ml Superflow Strep-Tacins sepharose, IBA, Göttingen, Germany) equilibrated with buffer W (100 mM Tris-HCl, 150 mM NaCl, 1 mM EDTA, 0.05% DDM, pH 8.0). After washing with 20 column volumes buffer W, Strep-tag membrane protein together with chemically cross-linked proteins were eluted with buffer E (100 mM Tris-HCl, 150 mM NaCl, 1 mM EDTA, 2.5 mM Desthiobiotin, 0.05% DDM, pH 8.0). Elution fractions were 10-fold concentrated using a centrifugal filter unit (Amicon YM10 filter device, Millipore). Elution fractions from samples co-expressing PapE were 20-fold concentrated. Samples were mixed with SDS-PAGE loading dye and split in two aliquots. For one aliquot of each sample cross-links between affinity-tag purified proteins and co-eluted interaction partners were separated by boiling samples for 20 min at 95°C. Subsequently, 30 µl from each sample were subjected to SDS-PAGE and separated proteins were electroblotted. Immunoblots were probed with antiserum against CpxA, CpxP, CpxR [25] and MalE (NEB). Immunodetection was carried out using SuperSignal West Pico Chemoluminescent Substrate ECL-kit (Thermo Scientific Pierce Protein Biology Products) with a peroxidase-conjugated anti-rabbit IgG antibody(GE Healthcare).

As a control for each experiment, entire cells were tested by immunoblotting with antibodies targeting CpxP and MalE (loading control) to verify sufficient protein expression.

### Cell fractionation

Cultures were grown in LB broth supplemented with appropriate antibiotics at 37°C. Cells were harvested at OD600 = 0.6 and fractionated as described with some minor modifications. [17]. In brief, cell pellets were normalized to the same Abs600 and suspended in Tris-Sucrose buffer (10 mM Tris-HCl buffer, pH 7.5; 0.5 M sucrose) and periplasmic fractions were generated by addition of buffer P2 (2 mg/ml lysozyme in 0.1 M EDTA, pH 7.5). After 30 min incubation at 4°C spheroplasts were collected by centrifugation (20,000×g for 20 min) and the supernatant was harvested as periplasmic fraction. Spheroplasts were resuspended in buffer P1 and disrupted by sonication (four pulses) on ice. Unbroken cells and cell debris were removed by centrifugation (7,000×g for 20 min). The supernatant was ultracentrifuged (100,000×g for 30 min) to pellet the membrane fraction. Fractions were analyzed by immune-blotting with antiserum to CpxA-Strep (IBA), CpxP (Pineda) or with antiserum to MalE (NEB) and detected with an ECL kit (GE Healthcare).

### Purification of proteins and preparation of proteoliposomes

CpxA-6His and 6His-CpxR were purified by Ni-affinity chromatography essentially as described [25]. CpxA-Strep purification followed the mSPINE procedure without formaldehyde treatment. Purified CpxA-6His and CpxA- Strep were incorporated into liposomes according to the established protocol [25].

### Phosphotransfer assay

Phosphotransfer from CpxA-His or CpxA-Strep incorporated in proteoliposomes to CpxR was assayed by Phos-tag acrylamide gel electrophoresis. For phosphotransfer assays, reconstituted CpxA-His or CpxA-Strep (5 µM) in buffer H (50 mM HEPES pH 7.5, 100 mM NaCl, 100 mM KCl) was mixed with CpxA (4 µM) and phosphorylation was started with 130 µM ATP and 5 µM MgCl_2_ at 30°C. Samples were withdrawn at 0, 1, 5, 10 and 20 minutes, mixed with sample buffer and stored on ice upon electrophoresis. Phospho-proteins were separated on a 10% acrylamide gel containing 50 µM Phos-tag™ acrylamide and 50 µM MnCl_2_. The gel was subsequently subjected to semi-dry Immuno-Blotting and immunoassayed by employing a CpxR-antibdy and chemiluminescence. Phosphorylated CpxR (indicated as CpxR∼P) mirates slower than non-phosphorylated CpxR and is accordingly seen as the upper band.

### β-Galactosidase Activity Analysis

β-Galactosidase assays were performed in strain SP594 [18] as described [56] with four biological replicates each with technical triplicates. For overproduction of CpxP and CpxPA108V, pTcpxP, pBcpxP and pBcpxPA108V plasmids were used [27].

### Analytical Methods

Protein concentrations were determined using the BCA Protein Assay Kit from Interchim (France) according to the manufacturer’s instructions.

## Supporting Information

Figure S1 BACTH shows that CpxP and the periplasmic sensor domain of CpxA can dimerize. BACTH experiments were performed as described in (Figure 1) using E. coli BTH101 co-transformed with plasmids encoding the different T25- and T18-hybrid proteins. T25- and T18-fragments fused to the leucine zipper of transcription factor GCN4 and the empty vectors served as positive (+) and negative (−) controls. (A) 3 µl of a LB overnight culture were spotted on a MacConkey-Lactose plate and incubated for 24 h at 30°C. (B) The degree of functional complementation between the indicated hybrid proteins was quantified by measuring ß-galactosidase activities in suspensions of toluene-treated E. coli BTH101 cells harboring the corresponding plasmids. The activity of the negative control (pKT25, pUT18C) represents the background (dashed line). Shown are the averages ± S.E.M. of three biological replicates each in technical triplicates (t test). Numbers above bars give percentage of ß-galactosidase activity relative to the positive control.
(TIF)

Figure S2 CpxA-Strep is active. (A) To check in which area of the cell CpxA-Strep is located, cell fractionation assays were performed. For this purpose *E. coli* TG1 cells producing CpxA-Strep (pKT01E) were grown in LB to OD600 = 0.6. Cells were harvested and periplasmic fractions (PF), membrane fractions (MF), cytosolic fractions (CF) and aggregated proteins derived from the low speed pellet (LSP) were prepared and subjected to immunological detection using antiserum to the Strep-tag, respectively. Purified CpxA-Strep served as control for antibody specificity (C). (B) To proof whether CpxA-Strep is active when purified according to the mSPINE protocol, phosphotransfer assays were performed using Phos-tag™ acrylamide. Therefore, CpxA-Strep and CpxA-His, respectively, were reconstituted (5 µM) into liposomes according to our established protocol [25]. CpxR was added (4 µM), phosphotransfer reaction was started by adding 130 µM ATP and 5 µM MgCl_2_ and incubated at 30°C. Samples were withdrawn at 0, 1, 5, 10 and 20 minutes, mixed with sample buffer and stored on ice upon electrophoresis. Positive control (PC) was created by phosphorylation of 4 µM CpxR wih 10 mM acetylphosphate for 20 minutes at 30°C as described in [59]. As negative control (NC) CpxR without phosphorylation reaction was used. Phospho-proteins were separated by Phos-tag™ acrylamide. The gel was subjected to semi-dry Western-Blotting and immune-assayed by employing a CpxR-antibody and chemiluminescence. Phosphorylated CpxR (indicated as CpxR∼P) migrates slower than non-phosphorylated CpxR.
(TIF)

Figure S3 pBcpxP allows inhibition of Cpx pathway. (A) Overexpression of *cpxP* from pBcpxP with 0.002% arabinose is sufficient to inhibit the Cpx-TCS as determined by promoter lacZ-fusion analysis using SP594 (PcpxP-lacZ). Shown are means ± S.E.M. of three independent experiments, each with two replicates. pBad33, pTrc99A and pTcpxP served as controls. (B) Cells from (A) were fractionated by spheroplast preparation and CpxP levels in periplasmic (P) and cytosolic (C) fractions were analysed by immunoblotting using antiserum to the CpxP protein, and the MalE protein (loading control), respectively. Purified, His6-CpxP and MalE served as controls for antibody specificity (K). Black triangles show specific and the white triangle unspecific reactions.
(TIF)

Figure S4 Induced expression of CpxP from pBadcpxP by 0.002% arabinose is sufficient to demonstrates physical interaction between CpxP and CpxA by Membrane-SPINE. mSPINE experiments were performed as described in (Figure 2A) with *E. coli* TG1 producing CpxA-Strep (pKT01E) and CpxP (pBcpxP) grown in LB supplemented with the indicated arabinose (A %) concentrations. Shown are representatives of two biological replicates. Again, CpxP is hardly detectable without overproduction (lane 2). Moreover, with increasing arabinose concentration the amount of captured CpxP increases (compare lane 8 with lanes 6 and 4). However, using high amount of arabinose to induce CpxP expression from the plasmid pBcpxP CpxP was also detectable in unboiled fractions indicating an excess of CpxP results in unspecific reactions. Strikingly, slight overproduction of CpxP from pBcpxP by 0.002% arabinose was sufficient to capture CpxP by CpxA-Strep enrichment (lane 8). Because no unspecific reactions were detectable for the unboiled fraction (lane 7) and samples without formaldehyde treatment (lane 10) we used for our further studies strains that slightly overproduced CpxP from pBcpxP by 0.002% arabinose.
(TIF)

Figure S5 The CpxPA108V inhibits the Cpx-two component system. (A) CpxP-dependent inhibition of the Cpx-TCS was determined by promoter lacZ-fusion analysis using SP594 (PcpxP-lacZ) producing CpxP or CpxPA108V from pBad33. Shown are means ± S.E.M. of three independent experiments, each with two replicates. pTrc99A and pTcpxP served as controls. (B) Cells from (A) were fractionated by spheroplast preparation and CpxP levels in periplasmic (P) and cytosolic (C) fractions were analysed by immunoblotting using antiserum to the CpxP protein, and the MalE protein (loading control), respectively. Purified, His6-CpxP and MalE served as controls for antibody specificity (K). Black triangles show specific and the white triangle unspecific reactions.
(TIF)

Figure S6 The N-terminal extension of PapE is critical for the induced release of CpxP from CpxA. A) mSPINE experiments were performed as described in Fig. 2A with *E. coli* TG1 producing CpxA-Strep, CpxP and the PapE variant PapEndt that misses the N-terminal extension important for Cpx system activation [39]. Purified CpxA-His6 and His6-CpxP served as controls for antibody specificity (K). Black triangles show specific and white triangles unspecific reactions. Shown are both biological dublicates. B) To visualize protein level in each mSPINE experiment, whole cells from (A) were collected after formaldehyde treatment, subjected to immunological determination using antiserum to CpxA, CpxP, and MalE (loading control). Purified CpxA-His6, His6-CpxP and MalE served as controls for antibody specificity (K). Black triangles show specific and white triangles unspecific reactions.
(TIF)
