# Supplementary figures and images for: Dynamic Interaction between the CpxA Sensor Kinase and the Periplasmic Accessory Protein CpxP Mediates Signal Recognition in E. coli
Source: PLoS One. 2014 Sep 10;9(9):e107383. doi: 10.1371/journal.pone.0107383 (PMC4160245; doi:10.1371/journal.pone.0107383)

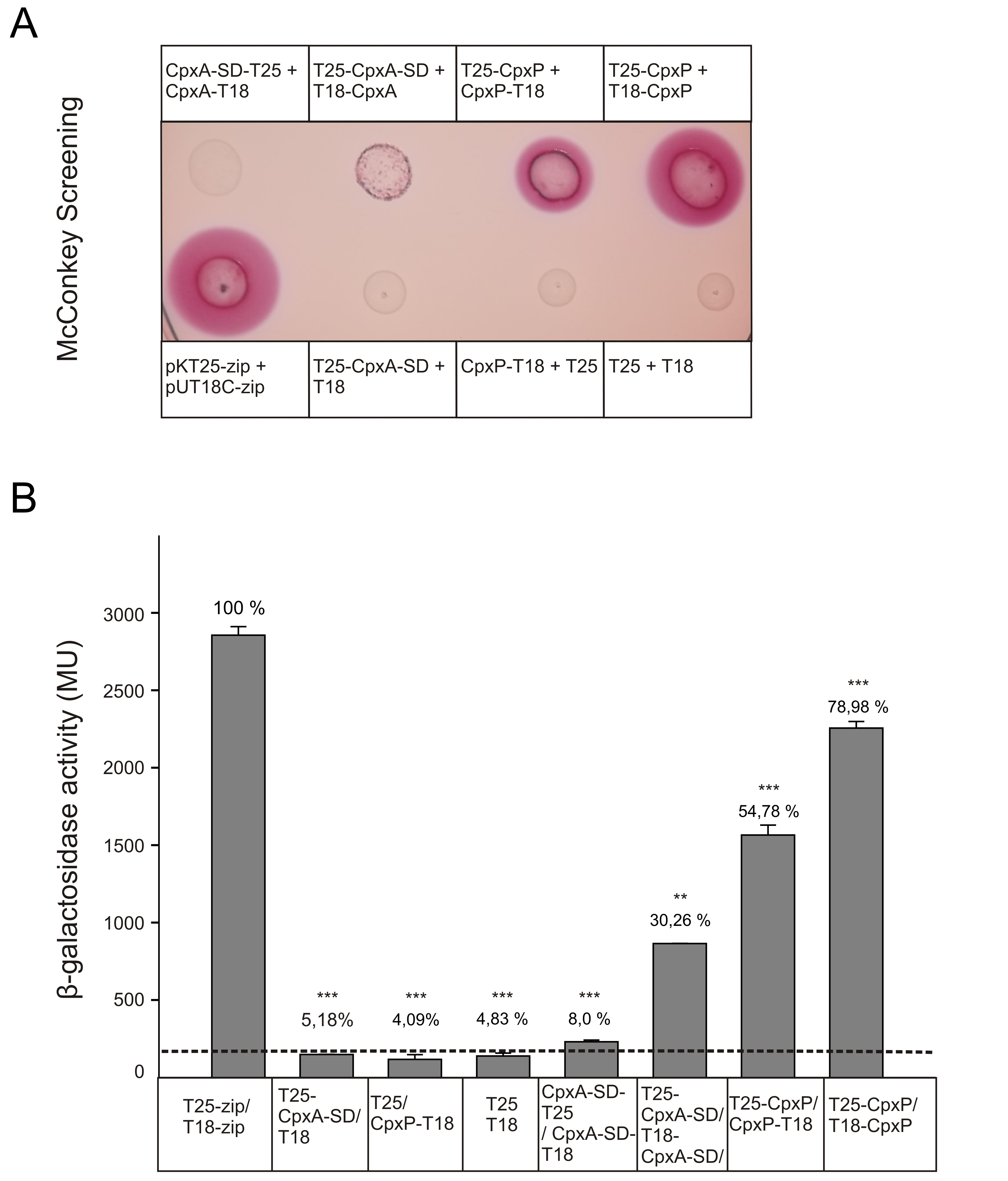

Supplement: Figure S1 — BACTH shows that CpxP and the periplasmic sensor domain of CpxA can dimerize. BACTH experiments were performed as described in (Figure 1) using E. coli BTH101 co-transformed with plasmids encoding the different T25- and T18-hybrid proteins. T25- and T18-fragments fused to the leucine zipper of transcription factor GCN4 and the empty vectors served as positive (+) and negative (−) controls. (A) 3 µl of a LB overnight culture were spotted on a MacConkey-Lactose plate and incubated for 24 h at 30°C. (B) The degree of functional complementation between the indicated hybrid proteins was quantified by measuring ß-galactosidase activities in suspensions of toluene-treated E. coli BTH101 cells harboring the corresponding plasmids. The activity of the negative control (pKT25, pUT18C) represents the background (dashed line). Shown are the averages ± S.E.M. of three biological replicates each in technical triplicates (t test). Numbers above bars give percentage of ß-galactosidase activity relative to the positive control. (TIF) [file pone.0107383.s001.tif]

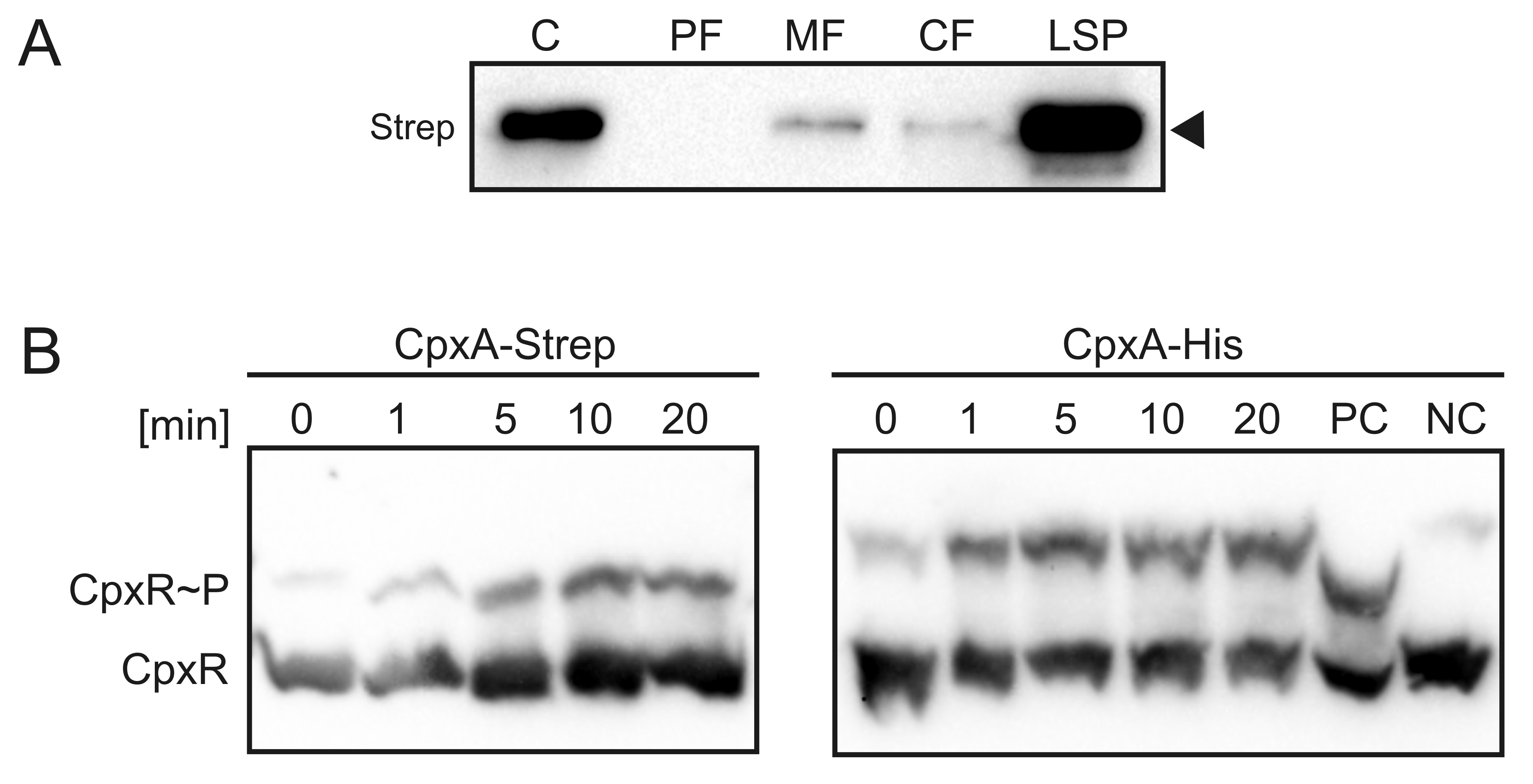

Supplement: Figure S2 — CpxA-Strep is active. (A) To check in which area of the cell CpxA-Strep is located, cell fractionation assays were performed. For this purpose E. coli TG1 cells producing CpxA-Strep (pKT01E) were grown in LB to OD600 = 0.6. Cells were harvested and periplasmic fractions (PF), membrane fractions (MF), cytosolic fractions (CF) and aggregated proteins derived from the low speed pellet (LSP) were prepared and subjected to immunological detection using antiserum to the Strep-tag, respectively. Purified CpxA-Strep served as control for antibody specificity (C). (B) To proof whether CpxA-Strep is active when purified according to the mSPINE protocol, phosphotransfer assays were performed using Phos-tag™ acrylamide. Therefore, CpxA-Strep and CpxA-His, respectively, were reconstituted (5 µM) into liposomes according to our established protocol [25]. CpxR was added (4 µM), phosphotransfer reaction was started by adding 130 µM ATP and 5 µM MgCl2 and incubated at 30°C. Samples were withdrawn at 0, 1, 5, 10 and 20 minutes, mixed with sample buffer and stored on ice upon electrophoresis. Positive control (PC) was created by phosphorylation of 4 µM CpxR wih 10 mM acetylphosphate for 20 minutes at 30°C as described in [59]. As negative control (NC) CpxR without phosphorylation reaction was used. Phospho-proteins were separated by Phos-tag™ acrylamide. The gel was subjected to semi-dry Western-Blotting and immune-assayed by employing a CpxR-antibody and chemiluminescence. Phosphorylated CpxR (indicated as CpxR∼P) migrates slower than non-phosphorylated CpxR. (TIF) [file pone.0107383.s002.tif]

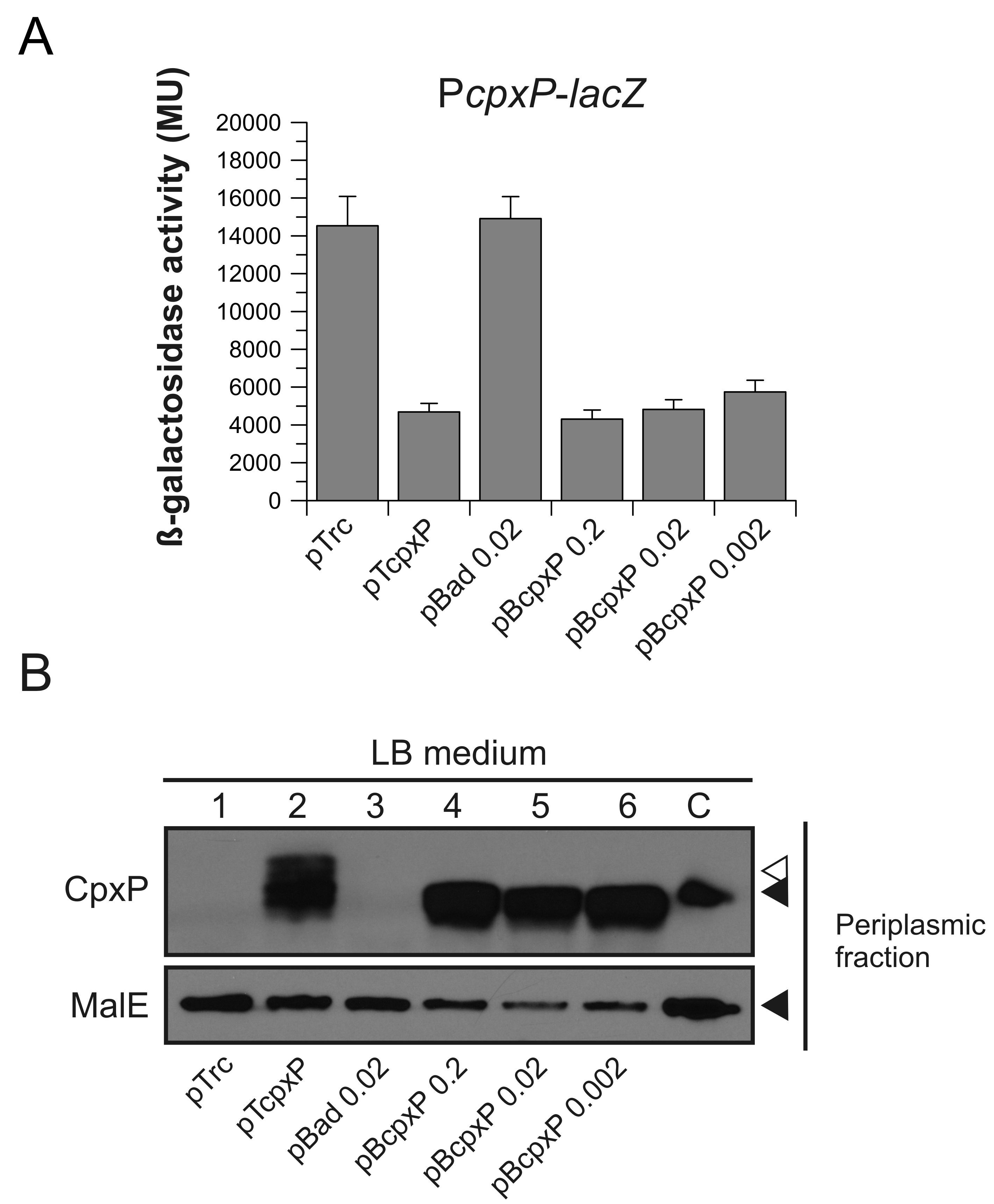

Supplement: Figure S3 — pBcpxP allows inhibition of Cpx pathway. (A) Overexpression of cpxP from pBcpxP with 0.002% arabinose is sufficient to inhibit the Cpx-TCS as determined by promoter lacZ-fusion analysis using SP594 (PcpxP-lacZ). Shown are means ± S.E.M. of three independent experiments, each with two replicates. pBad33, pTrc99A and pTcpxP served as controls. (B) Cells from (A) were fractionated by spheroplast preparation and CpxP levels in periplasmic (P) and cytosolic (C) fractions were analysed by immunoblotting using antiserum to the CpxP protein, and the MalE protein (loading control), respectively. Purified, His6-CpxP and MalE served as controls for antibody specificity (K). Black triangles show specific and the white triangle unspecific reactions. (TIF) [file pone.0107383.s003.tif]

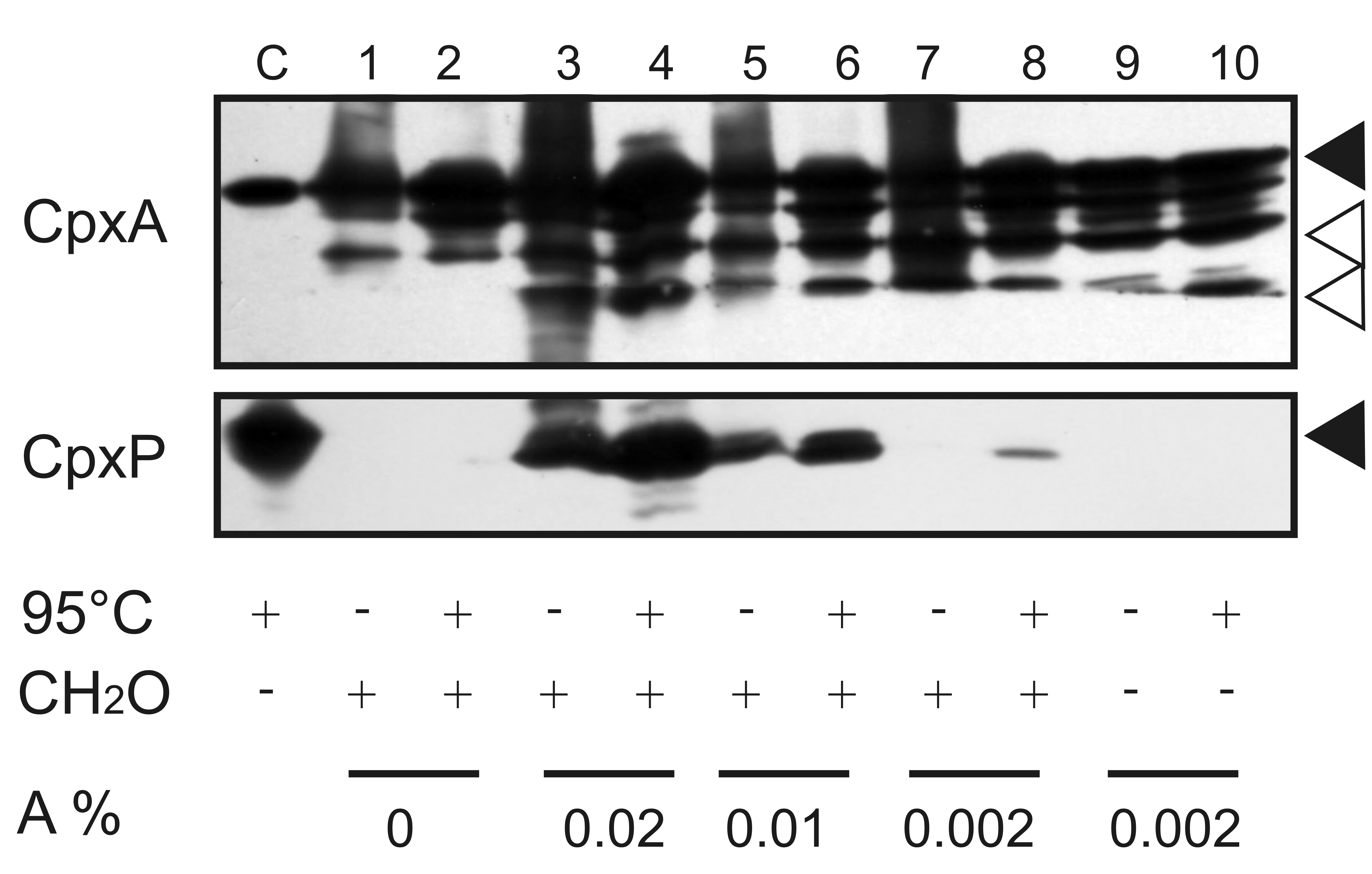

Supplement: Figure S4 — Induced expression of CpxP from pBadcpxP by 0.002% arabinose is sufficient to demonstrates physical interaction between CpxP and CpxA by Membrane-SPINE. mSPINE experiments were performed as described in (Figure 2A) with E. coli TG1 producing CpxA-Strep (pKT01E) and CpxP (pBcpxP) grown in LB supplemented with the indicated arabinose (A %) concentrations. Shown are representatives of two biological replicates. Again, CpxP is hardly detectable without overproduction (lane 2). Moreover, with increasing arabinose concentration the amount of captured CpxP increases (compare lane 8 with lanes 6 and 4). However, using high amount of arabinose to induce CpxP expression from the plasmid pBcpxP CpxP was also detectable in unboiled fractions indicating an excess of CpxP results in unspecific reactions. Strikingly, slight overproduction of CpxP from pBcpxP by 0.002% arabinose was sufficient to capture CpxP by CpxA-Strep enrichment (lane 8). Because no unspecific reactions were detectable for the unboiled fraction (lane 7) and samples without formaldehyde treatment (lane 10) we used for our further studies strains that slightly overproduced CpxP from pBcpxP by 0.002% arabinose. (TIF) [file pone.0107383.s004.tif]

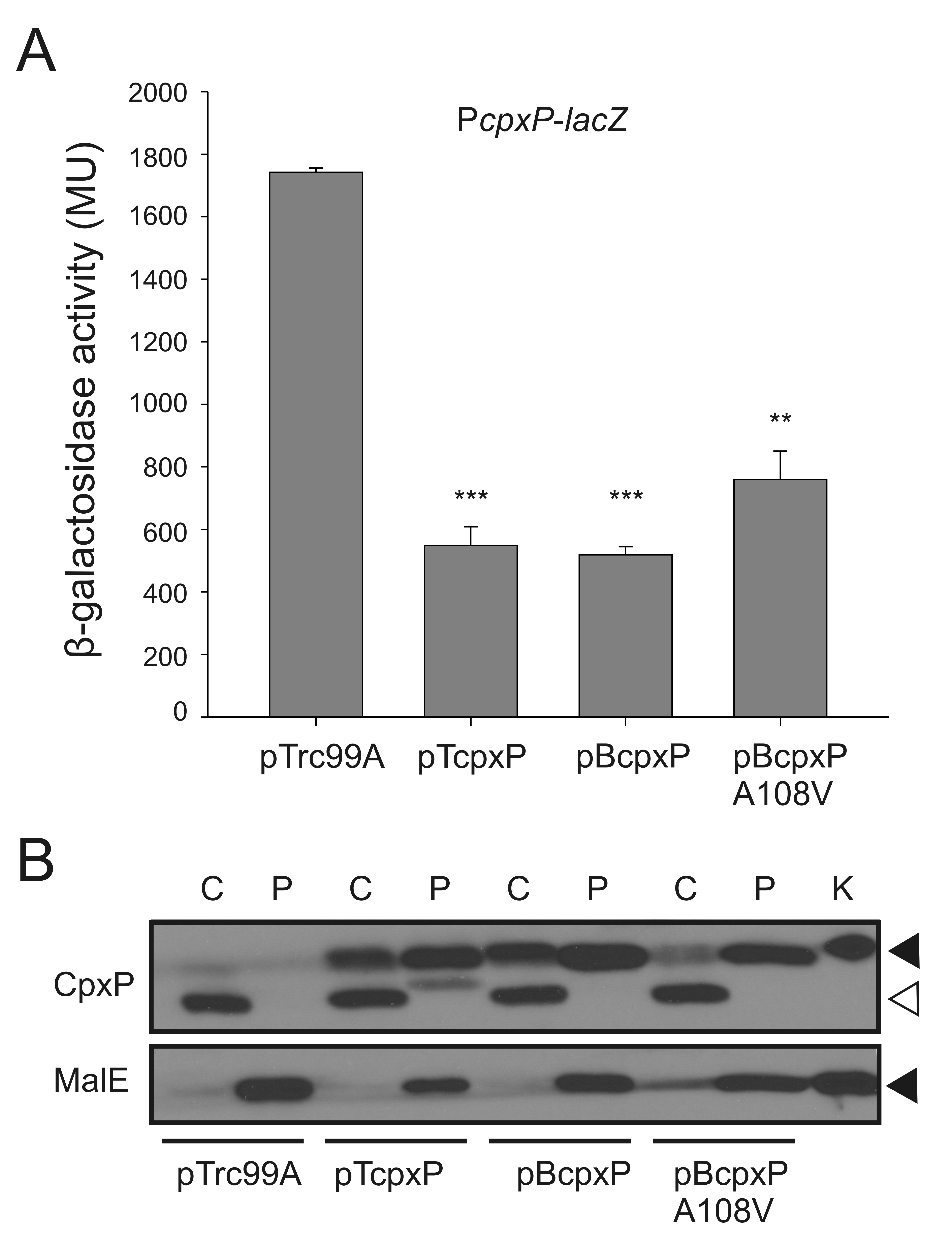

Supplement: Figure S5 — The CpxPA108V inhibits the Cpx-two component system. (A) CpxP-dependent inhibition of the Cpx-TCS was determined by promoter lacZ-fusion analysis using SP594 (PcpxP-lacZ) producing CpxP or CpxPA108V from pBad33. Shown are means ± S.E.M. of three independent experiments, each with two replicates. pTrc99A and pTcpxP served as controls. (B) Cells from (A) were fractionated by spheroplast preparation and CpxP levels in periplasmic (P) and cytosolic (C) fractions were analysed by immunoblotting using antiserum to the CpxP protein, and the MalE protein (loading control), respectively. Purified, His6-CpxP and MalE served as controls for antibody specificity (K). Black triangles show specific and the white triangle unspecific reactions. (TIF) [file pone.0107383.s005.tif]

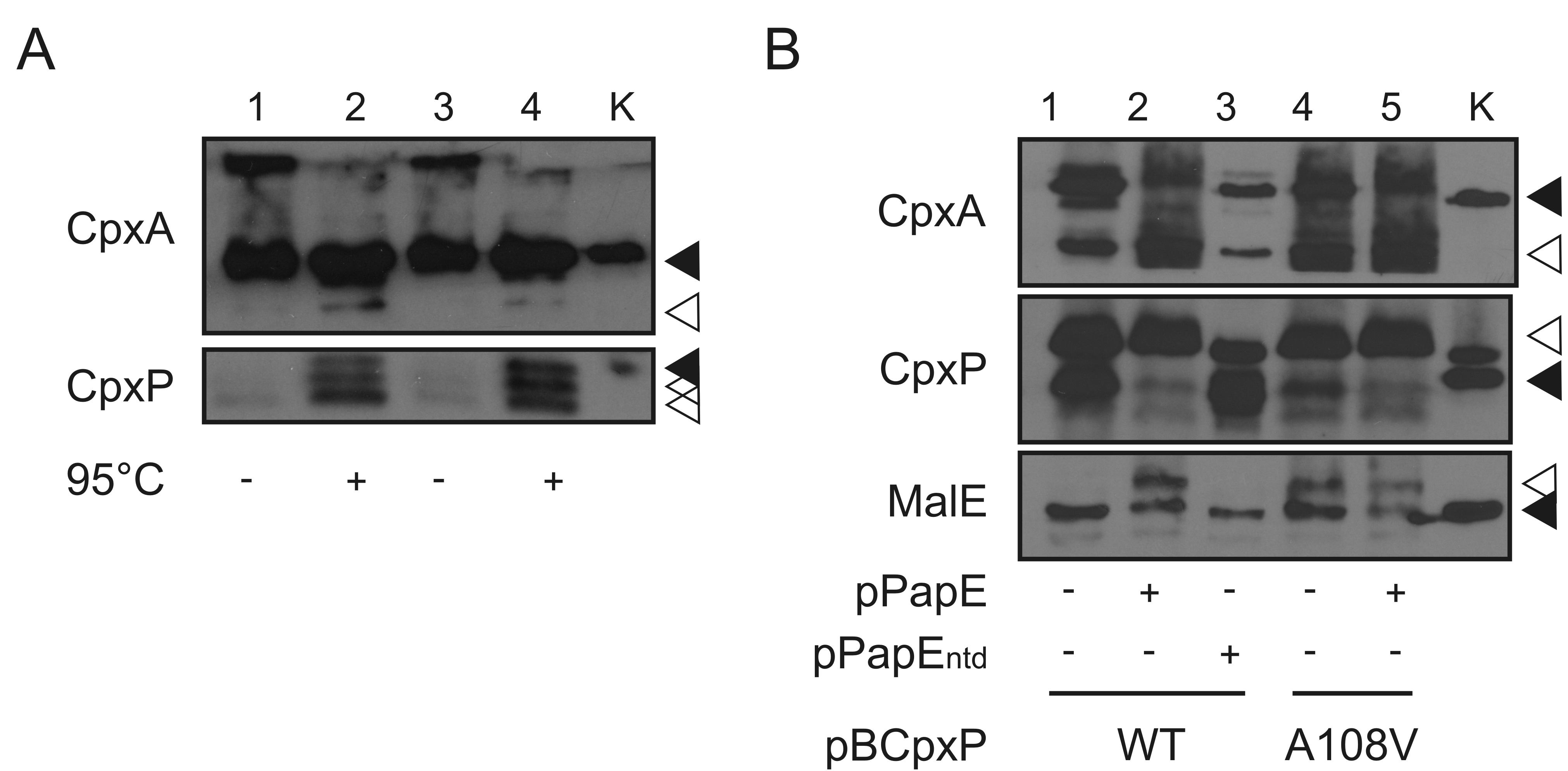

Supplement: Figure S6 — The N-terminal extension of PapE is critical for the induced release of CpxP from CpxA. A) mSPINE experiments were performed as described in Fig. 2A with E. coli TG1 producing CpxA-Strep, CpxP and the PapE variant PapEndt that misses the N-terminal extension important for Cpx system activation [39]. Purified CpxA-His6 and His6-CpxP served as controls for antibody specificity (K). Black triangles show specific and white triangles unspecific reactions. Shown are both biological dublicates. B) To visualize protein level in each mSPINE experiment, whole cells from (A) were collected after formaldehyde treatment, subjected to immunological determination using antiserum to CpxA, CpxP, and MalE (loading control). Purified CpxA-His6, His6-CpxP and MalE served as controls for antibody specificity (K). Black triangles show specific and white triangles unspecific reactions. (TIF) [file pone.0107383.s006.tif]
